# Progress in the research of thunder-fire moxibustion and warm acupunct ure-moxibustion combined with western medicine in treating knee osteoarthritis

**DOI:** 10.12669/pjms.41.6.10621

**Published:** 2025-06

**Authors:** Fan Zhang, Dianhui Yang

**Affiliations:** 1 Fan Zhang College of Acupuncture-moxibustion and Tuina, Shandong University of Traditional Chinese Medicine, Jinan 250355, Shandong, China; 2 Dianhui Yang College of Acupuncture-moxibustion and Tuina, Shandong University of Traditional Chinese Medicine, Jinan 250355, Shandong, China. Department of Acupuncture-moxibustion, The Affiliated Hospital of Shandong University of Traditional Chinese Medicine, Jinan 250014, Shandong, China

**Keywords:** Integrated traditional Chinese and western medicine, Knee osteoarthritis, Thunder-fire moxibustion, Warm acupuncture-moxibustion

## Abstract

**Objective:**

To summarize the research progress of thunder fire moxibustion and warm acupuncture and moxibustion in treating knee osteoarthritis in world during the period 2020-2024.

**Methods:**

This is a descriptive research. We recorded and evaluated the literature on the treatment of knee osteoarthritis with thunder fire moxibustion and warm acupuncture and moxibustion published on the Science Information Network and the National Institute of Knowledge Infrastructure of China during 2020-2024. This paper probes into the mechanism of thunder fire moxibustion and warm acupuncture and moxibustion in treating knee osteoarthritis, and evaluates the clinical efficacy of thunder fire moxibustion and warm acupuncture and moxibustion in treating knee osteoarthritis.

**Results:**

In clinical practice, thunder fire moxibustion and warm acupuncture and moxibustion can play a synergistic role in assisting western medicine. The combination of traditional and western medicine has complementary advantages, which can alleviate pain, improve the function of the knee joint, and has high safety, improving the overall efficacy of knee osteoarthritis treatment.

**Conclusions:**

Thunder fire moxibustion and warm acupuncture and moxibustion have ideal efficacy and high safety in the treatment of knee osteoarthritis. In the future, it is still necessary to explore personalized treatment schemes, combining thunder fire moxibustion, warm acupuncture and moxibustion with modern technologies such as physical therapy and drug therapy, so as to achieve personalized and precise treatment.

## INTRODUCTION

Knee osteoarthritis is a chronic joint disease featuring pathological changes such as articular cartilage degeneration, destruction and hyperosteogeny in most cases, which seriously affects the quality of life of patients. Thunder-fire moxibustion and warm acupuncture-moxibustion are two major therapeutic options in traditional Chinese medicine(TCM) with definite curative effects for treating knee osteoarthritis. The two methods exhibit the advantages of pain relief, knee joint function improvement, and high safety. In clinical practice, thunder-fire moxibustion and warm acupuncture-moxibustion combined with western medicine can play a synergistic role in treating knee osteoarthritis. The combined therapy can promote the complementary advantages of TCM and western medicine to improve the overall curative effect of patients eventually. Knee osteoarthritis belongs to the category of “bi syndrome” and “bone bi” in TCM,^1^ with symptoms mainly including joint pain, joint stiffness, and joint dysfunction. The clinical incidence of this disease is high, and it tends to occur in middle-aged and elderly people. It needs timely treatment, otherwise it will affect the quality of life of patients. The main methods for treating knee joints in Western medicine are medication and surgery.^2^ Western medicine treatment can alleviate pain and inhibit inflammation, but long-term use has significant side effects. Although surgical treatment is effective, it is not suitable for some patients. In recent years, TCM has made significant progress in treating knee osteoarthritis, which can make up for the shortcomings of Western medicine treatment. TCM believes that knee osteoarthritis belongs to the categories of “bi syndrome” and “bone bi”,^3^ mainly caused by liver and kidney deficiency due to insufficient qi and blood, and obstruction of qi caused by invasion of wind cold dampness pathogens. Thunder fire moxibustion and warm needle moxibustion are common Chinese medicine techniques. Thunder fire moxibustion, as a special moxibustion method,^4^ is a combination of traditional Chinese medicine and moxibustion principles. Through the burning of special pharmaceutical moxa sticks, heat can penetrate into the skin, warm the meridians, dispel wind and dissipate cold. Warm acupuncture and moxibustion combines acupuncture and moxibustion to stretch tendons, activate collaterals, and harmonize qi and blood.

However, so far, there is still a lack of systematic summary and analysis on this topic. As such this study aimed to summarize the research progress of thunder fire moxibustion and warm acupuncture and moxibustion in treating knee osteoarthritis in world during the period 2020-2024, as well as treatment mechanisms of the two therapeutic approaches. It is expected to provide potential reference for the treatment of knee osteoarthritis in TCM.

## METHODS

### Pathogenesis and treatment of knee osteoarthritis in western medicine:

The pathogenesis of knee osteoarthritis in western medicine shows a close relationship with degenerative changes in articular cartilage, dysimmunity, environment, climate, etc. Specifically, older populations are at high risk of developing knee osteoarthritis since they are prone to developing articular cartilage degeneration that may result in wear and thinning of the articular cartilage. Knee osteoarthritis is also common in obese patients, and those with long-term overuse or strain of joints. Dysimmunity can also accelerate the destruction of cartilage, thereby triggering inflammatory reactions in joints. Besides, the living and climatic conditions of patients are also associated with joint health. For instance, patients living in a cold and shady environment may experience decreased blood flow velocity locally, leading to the aggravation of knee osteoarthritis.

At present, multiple therapeutic approaches in western medicine are available for the treatment of knee osteoarthritis, mainly including medication, intra-articular injection, surgical treatment, etc. Among them, non-steroidal anti-inflammatory drugs are commonly used for medication, which can effectively alleviate pain and inflammation. In a study carried out by Kim MS et al.^4^, celecoxib could improve symptoms and alleviate pain to some extent in patients with knee osteoarthritis. Nevertheless, the overall therapeutic effect cannot be ensured when using medication alone as it may cause adverse reactions in the gastrointestinal and cardiovascular systems. Furthermore, surgery is effective for patients with severe knee osteoarthritis. Zhang AR et al.^5^ reported that total knee arthroplasty was effective for patients with advanced knee osteoarthritis to relieve pain and restore joint function. However, the effect of surgery may b e compromised owing to its great surgical trauma, multiple postoperative complications, and prolonged recovery period. Significantly, with significant progress achieved in the treatment of knee osteoarthritis recently, TCM can serve as an important supplementary means for western medicine.

### Pathogenesis and treatment of knee osteoarthritis in TCM:

According to traditional medicine, knee osteoarthritis belongs to the categories of “Bi syndrome”, “arthralgia”, etc., and is more common in the elderly with physical deficiency, as well as those with Qi-blood insufficiency, and liver-kidney deficiency.^1^ Due to the deficiency of vial Qi, exogenous pathogenic factors (e.g., wind, cold, and dampness) may invade the human body easily, which may further intersect with endogenous pathological factors, resulting in Qi-blood obstruction around the joints and disordered circulation. The obstruction may further induce pain; failure of blood to nourish tendons and bones may cause muscle and bone stiffness; and disordered Qi-blood may induce immobility.

Traditional Chinese medicine treatment of “Bi syndrome” generally follows a holistic concept, with particular emphasis placed on treatment with syndrome differentiation. Various therapeutic methods such as acupuncture and moxibustion, massage and medical treatment of TCM can be adopted according to patients’ conditions. To be specific, by stimulating meridians and acupoints, acupuncture and moxibustion can regulate Qi-blood circulation, relax tendons and unblock meridians to smooth Qi-blood circulation of arthralgia, thus relieving pain and restoring joint function.

Meanwhile, by applying professional techniques, massage of muscles and tendons of joints can promote blood circulation, remove blood stasis, relax tendons, and relieve spasms, leading to unobstructed Qi-blood circulation to reduce pain. Medical treatment of TCM depends on the individual involved by following the principle of treatment with syndrome differentiation. For instance, for patients with arthralgia due to wind-cold-dampness, medicines with effects of warming meridians, dispersing cold, dispelling wind and removing dampness are used to expel exogenous pathogenic factors; while for those with liver-kidney deficiency, medicines enhancing Qi-blood circulation, strengthening muscles and bones are applied to promote the relief of arthralgia voluntarily.

### Research progress on thunder-fire moxibustion for the treatment of knee osteoarthritis:

Effect and mechanism of thunder-fire moxibustion for the treatment of knee osteoarthritis. Thunder-fire moxibustion is a traditional moxibustion therapy using TCM powder and moxa.^6^ Based on the theory of meridians and collaterals, thunder-fire moxibustion also integrates modern medical knowledge to form a unique treatment system. It is developed and innovated from the ancient “thunder-fire miraculous needle”, with its original formula and usage methods changed to meet the needs of the current generations. During thunder-fire moxibustion, the heat generated by burning drugs can stimulate relevant acupoints through suspended moxibustion. The thermal effect of moxibustion can stimulate meridian-Qi to get through the grain of the skin and the texture of the subcutaneous flesh so that drugs can act on corresponding acupoints.

Meanwhile, the TCM substances of thunder-fire moxibustion can produce active ingredients in the process of moxibustion, which can further penetrate into the body through the unobstructed grain of skin and the texture of the subcutaneous flesh, and transmit along the meridians, achieving the effect of dredging meridians, promoting blood circulation, and removing blood stasis.^7^ For example, Huang Q et al.^8^ reported that thunder-fire moxibustion alone is effective for patients with mild to moderate knee osteoarthritis, which can significantly alleviate pain and promote function repair of patients’ knee joints. Effect and mechanism of thunder-fire moxibustion-based combined therapies for the treatment of knee osteoarthritis. Thunder-fire moxibustion alone has a unique therapeutic effect, which, however, may be ineffective for patients with severe knee osteoarthritis, requiring combined application with other therapies to achieve excellent therapeutic effects.

Zhao J et al.^9^ revealed that thunder-fire moxibustion combined with Duhuo Jisheng Decoction can improve the overall response rate of patients. Guo Z et al.^10^ found in their research that thunder-fire moxibustion combined with ultrashort wave therapy can effectively promote knee joint function repair in patients with knee osteoarthritis. Zeng L et al.^11^ also supported that thunder-fire moxibustion combined with acupoint application can exert a synergistic effect in treating knee osteoarthritis, which can improve patients’ oxidative stress. Collectively, all these findings suggest that thunder-fire moxibustion combined with other therapies do have significant advantages in treating knee osteoarthritis.^12^-^14^

Firstly, thunder-fire moxibustion itself can warm and unblock meridians, and promote Qi-blood circulation, which can improve local blood circulation in the knee joint to provide a favorable microenvironment for tissue repair. It can improve the overall response rate of patients when applied jointly with Duhuo Jisheng Decoction, demonstrating the effect of integrating TCM and western medicine. Secondly, thunder-fire moxibustion combined with ultrashort wave therapy can effectively promote knee joint function repair in patients with knee osteoarthritis, uncovering an enhanced therapeutic effect of TCM and physical therapy. In addition, besides a synergistic effect, the combined use of thunder-fire moxibustion and acupoint application can also improve patients’ oxidative stress and the health of their knee joints.

### Research progress on warm acupuncture-moxibustion for the treatment of knee osteoarthritis:

Effect and mechanism of warm acupuncture-moxibustion for the treatment of knee osteoarthritis shows that by using acupuncture and moxibustion jointly in warm acupuncture-moxibustion, various meridians and acupoints can be stimulated by twirling, lifting and thrusting the acupuncture needle inserted into the acupoints to achieve the purpose of treatment. It also exerts a therapeutic role based on the warming effect of moxibustion by igniting the moxa or moxa stick on the needle bat.^15^ Mechanisms related to its therapeutic effect are described in detail as follows:

### Warm and activate meridians:

this therapy adopts moxibustion to exert a warming effect and acupuncture to stimulate meridians and acupoints jointly, which can warm and unblock meridians to relieve pain and treat other diseases;

### Invigorate Qi-blood circulation:

acupuncture and moxibustion can work synthetically to promote Qi-blood circulation in the body, and dissipate stagnant blood;^16^

Regulate Yin and Yang: through this intervention, specific acupoints can be stimulated to balance internal Yin-Yang and prevent Yin-Yang imbalance, thus improving Bi syndrome; and

### Strengthen the body resistance to eliminate pathogenic factors:

warming acupuncture-moxibustion can strengthen the body’s resistance to diseases by strengthening the body resistance to eliminate pathogenic factors. In a study by Zhao L et al.^17^, compared with carbon dioxide laser moxibustion, warm acupuncture-moxibustion was more effective in treating patients with mild to moderate knee osteoarthritis, which could significantly reduce serum levels of inflammatory factors, and improve patients’ activity of daily living. It further supports the effect of warm acupuncture-moxibustion for patients with mild to moderate knee osteoarthritis.

### Effect and mechanism of warm acupuncture-moxibustion-based combined therapies:

According to a study carried out by Sun Z et al.^18^, after treatment using meloxicam combined with warm acupuncture-moxibustion, patients had improved overall response rate and reduced expressions of inflammatory factors. It suggests the efficacy and safety of warm acupuncture-moxibustion combined with western medicine in the treatment of patients with knee osteoarthritis, which can improve clinical symptoms, and repair injured soft tissues of knee joints. It can be speculated that by warming and activating meridian, and invigorating Qi-blood circulation, warm acupuncture-moxibustion can stimulate local blood circulation to benefit inflammation resolution and tissue repair; simultaneously, the use of western medicine jointly can relieve pain and reduce inflammation rapidly.

Eventually, combined use of the two methods can quickly alleviate clinical symptoms, and improve therapeutic effect and safety. Furthermore, Liu ZF et al.^19^ also discovered in their research that warm acupuncture-moxibustion combined with massage can effectively improve the pain score and swelling score of patients, and relieve their signs and symptoms. It indicates that warm acupuncture-moxibustion combined with other TCM therapies can improve the overall efficacy of individual therapy and improve symptoms. It can be attributed to the effect of warm acupuncture-moxibustion on stimulating acupoints, and that of massage on relaxing muscles and fascia. The combined use can contribute to improved symptoms from different perspectives to realize a more comprehensive effect.

In addition, Yin X et al.^20^ revealed that a blade needle combined with warm acupuncture-moxibustion can improve the overall response rate of patients with knee osteoarthritis, up to 90.00%, supporting the role of the proposed therapy in improving the curative effect of knee osteoarthritis. As for the cause, a blade needle is minimally invasive and safe to relieve the excessive stress of the injured soft tissue, and release the adhesive soft tissue. The combined application of a blade needle and warm acupuncture-moxibustion exhibits a definite curative effect synergistically.

## PROSPECT

It is expected that with the progress of science and technology, future research can be performed actively to reveal the therapeutic mechanisms of the two therapies, and explore their impact on the pathological conditions of patients based on molecular biology, genome biology and other technologies to provide clinical evidence. Meanwhile, further research can be conducted to actively explore personalized therapeutic schedules based on patients’ physical constitutions and pathological conditions, and accurately analyze pathological conditions through artificial intelligence and big data models, so as to achieve personalized and precise treatment.

In the future, there is a need to emphasize technological innovation and integration, and integrate thunder-fire moxibustion, warm acupuncture-moxibustion with physical therapy, medication and other modern technologies to achieve desired therapeutic effects. It is expected that the proposed interdisciplinary integration and innovation can contribute to more comprehensive and safer treatment for patients with knee osteoarthritis.

### Limitations:

It only includes relevant literature from one domestic database in China, and excludes conference reports, case reports, etc. Further discussion and analysis are needed to expand the scope of the database.

## CONCLUSIONS

Thunder-fire moxibustion can effectively alleviate pain and movement disorders in patients with knee osteoarthritis on the basis of its unique advantages in dispelling cold and promoting Qi circulation to remove the meridian obstruction. Warm acupuncture-moxibustion integrates the dual advantages of acupuncture and moxibustion to promote Qi-blood circulation by stimulating specific acupoints, thus harmonizing Yin-Yang, and strengthening the body resistance to eliminate pathogenic factors. Collectively, thunder-fire moxibustion and warm acupuncture-moxibustion are effective and safe in the treatment of knee osteoarthritis.

### Authors’ Contributions:

**FZ:** Literature search, Drafted the manuscript.

**DY:** Is responsible and accountable for the accuracy or integrity of the work.

All authors have read and approved the final manuscript.
